# The applications of TMS in brain function assessment and treatment of mental disorders: a narrative review

**DOI:** 10.3389/fpsyt.2026.1860185

**Published:** 2026-07-06

**Authors:** Youyou Teng, Ziqi Gu, Ling Ding, Huicong Shi, Zhifei Gao, Haiyun Xu

**Affiliations:** 1 The Affiliated Wenzhou Kangning Hospital, School of Mental Health, Wenzhou Medical University, Wenzhou, Zhejiang, China; 2 Center for Sleep and Circadian Medicine, The Affiliated Brain Hospital, Guangzhou Medical University, Guangzhou, Guangdong, China; 3 Peking University Sixth Hospital, Peking University Institute of Mental Health, National Health Center (NHC) Key Laboratory of Mental Health (Peking University), National Clinical Research Center for Mental Disorders (Peking University Sixth Hospital), Beijing, China; 4 Department of Rehabilitation Medicine, Second Affiliated Hospital and Yuying Children’s Hospital of Wenzhou Medical University, Wenzhou, Zhejiang, China; 5 Department of Neurology, The Second Affiliated Hospital, School of Medicine, Zhejiang University, Hangzhou, Zhejiang, China

**Keywords:** brain function assessment, brain neuroplasticity, mental disorders, monoamines, rTMS

## Abstract

Over the past several decades, monoaminergic system dysfunction has been considered a key factor in the pathophysiology of some mental disorders. However, the therapeutic efficacy of drug therapy based on the monoamine hypothesis is unsatisfactory. In 2008, FDA approved the application of the repetitive transcranial magnetic stimulation (rTMS) for the treatment of patients with depression. Since then, TMS as a non-invasive neuromodulation technique, has been widely used to treat patients with mental disorders and assess their brain functions. This review article outlined the stimulation modes and parameter settings of TMS, summarized the therapeutic effects of TMS on main mental disorders such as depression, bipolar disorder (BD), schizophrenia, attention deficit/hyperactivity disorder (ADHD), and autism spectrum disorder (ASD), and synthesized the cellular and molecular mechanisms of its therapeutic effects for the mental disorders mainly based on preclinical research. These mechanisms involve pathophysiological processes such as neurotransmitter expression, neuroinflammation, neurotrophic factor production, oxidative stress, and gene expression related to brain plasticity and apoptosis in the brain. Furthermore, it introduced the emerging applications that combine TMS with other non-invasive neuroimaging techniques to assess brain function, including mapping neural pathways, measuring cortical excitability, and evaluating brain neuroplasticity. These advances have facilitated real-time evaluation of the therapeutic efficacy of TMS and optimized its application in treating mental disorders.

## Introduction

Over the past a couple of decades, advancements in neuroscience have linked neurotransmitter dysfunction to mental disorders, epitomized by the so-called monoamine hypothesis. This hypothesis posits that the clinical manifestations of major psychiatric disorders such as depression, anxiety, schizophrenia, and attention deficit hyperactivity disorder (ADHD) stem from imbalances in monoamine substances in the brain, including serotonin (5-HT), dopamine (DA), and norepinephrine (NE). In support of the monoamine hypotheses, chlorpromazine, the first antipsychotic drug, acts on DA receptors in the brain, while monoamine oxidase (MAO) inhibitors, such as moclobemide, alleviate depressive symptoms by inhibiting activities of MAO enzymes (1, 2). However, the monoamine hypothesis has not been fully validated in patients, partly due to the lack of non-invasive techniques that can measure the distribution and levels of neurotransmitters in the brain with high spatial and temporal resolution. Moreover, the efficacy of drug therapy based on the monoamine hypothesis is unsatisfactory. At least 50% of patients with depression do not respond to first-line antidepressants, and approximately 20% of patients with schizophrenia show little improvement with monotherapy of antipsychotics (3, 4).

**Figure 1:**
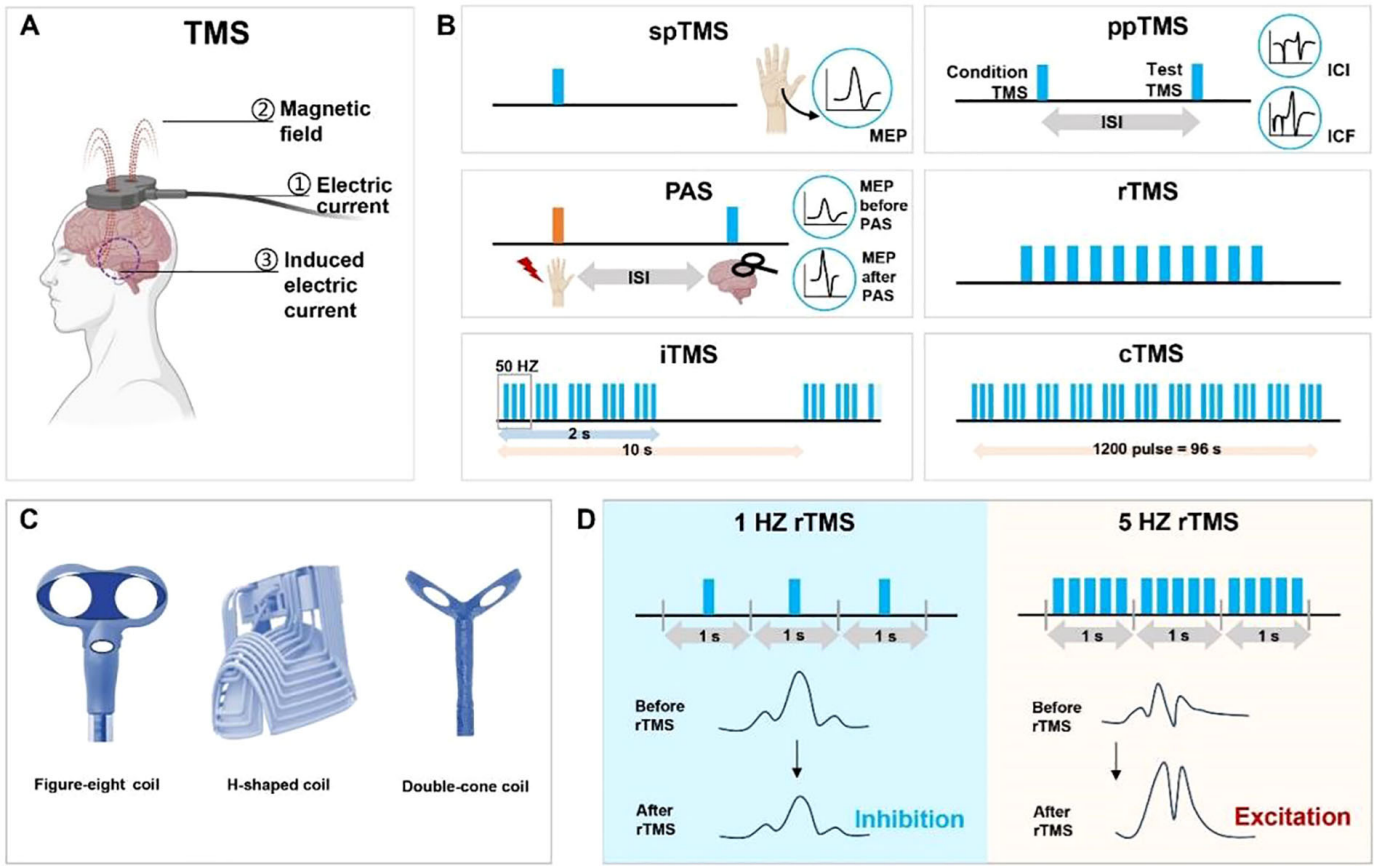
TMS stimulation patterns and parameters. **(A)** The model diagram shows the basic principle of TMS. **(B)** The different stimulation modes of TMS include spTMS, ppTMS, PAS, rTMS, iTMS and cTMS. **(C)** Coil shapes for TMS. Model diagrams of a figure-eight coil, an H-shaped coil, and a biconical coil are shown. **(D)** TMS produces different effects with different stimulation frequencies. cTMS, continuous transcranial magnetic stimulation; iTMS, integrated transcranial magnetic stimulation; PAS, paired associative stimulation; ppTMS, paired-pulse transcranial magnetic stimulation; rTMS, repetitive transcranial magnetic stimulation; spTMS, single-pulse transcranial magnetic stimulation; TMS, transcranial magnetic stimulation.

While pharmacological treatments for mental disorders have stalled, non-invasive physical interventions and diagnostic technologies, such as structural magnetic resonance imaging (sMRI), functional MRI (fMRI), diffusion tensor imaging (DTI), magnetic resonance spectroscopy (MRS), positron emission tomography (PET), single photon emission (SPECT), and transcranial magnetic stimulation (TMS), have ushered in a new era (5–8). Of these new techniques, TMS deserves special attention here. Unlike pure neuroimaging-based assessment techniques such as sMRI, fMRI, and PET that passively detect structural, hemodynamic, or metabolic signals, TMS influences neuronal activity by stimulating or inhibiting targeted brain regions through a weak current induced by pulsed magnetic fields from the TMS device (**Figure 1A**).

TMS is used both as an assessment tool to examine brain function and as a non-invasive treatment for various mental disorders, particularly when other methods like medication and psychotherapy have not been effective. The advantages of TMS include: 1) it is non-invasive; 2) it can assess and modulate concurrently neural excitability and plasticity; 3) it has fewer side effects than pharmacotherapy; and 4) there are a variety of combinations of it with other neural analysis and modulation techniques. This article aims to introduce the applications of TMS in evaluating brain functions and treating major mental disorders. It starts with the introduction of stimulation patterns and parameters of TMS employed in clinical and preclinical applications, then summarizes the applications of TMS in brain function assessment, followed by therapeutic effects of TMS on mental disorders including major depressive disorder (MDD), bipolar disorder (BD), schizophrenia, attention-deficit/hyperactivity disorder (ADHD), and autism spectrum disorder (ASD). Before the concluding remarks, we synthesize the neurobiological and molecular mechanisms underlying the therapeutic effects of TMS on mental disorders.

## TMS stimulation patterns and parameters

### Stimulation patterns

A variety of stimulation modes/patterns of TMS are being used in clinical practice and research, each with unique clinical applications tailored to specific diagnostic and therapeutic needs. Single pulse TMS (spTMS) targets the cerebral cortex, where motor neurons are stimulated, and the effect is transmitted to target muscles thus producing motor-evoked potentials (MEPs). This mode is often used to assess the integrity of the corticospinal tract. Paired-pulse TMS (ppTMS) delivers two sequential pulses at specific time intervals to produce neurophysiological responses within the same or both cerebral hemispheres. It is often used to assess excitatory and inhibitory interactions within or between hemispheres (9). Paired associative stimulation (PAS) combines peripheral nerve stimulation with cortical TMS, making it a valuable tool for studying synaptic plasticity (10). The repetitive transcranial magnetic stimulation (rTMS) involves the continuous delivery of pulses at fixed intensities and frequencies to a targeted brain region. It is widely used to treat various clinical conditions, particularly in neuropsychiatry. Theta burst stimulation (TBS) uses clusters of three biphasic pulses delivered at high (50 Hz) and low (5 Hz) frequencies. This mode is gaining attention for its therapeutic potential. Within TBS, intermittent TBS (iTBS) enhances cortical excitability while continuous TBS (cTBS) suppresses it. These different stimulation modalities expand the versatility of TMS, allowing both detailed neurological assessment and innovative therapeutic applications (**Figure 1B**).

### Coil shape and orientation

A key component of TMS equipment is its various shaped coils. According to the Faraday’s Law of Electromagnetic Induction, the alternating current in the coil located above the head will induce an alternating magnetic field, which non-invasively penetrates the skull and soft tissue below, and induces weak currents acting on cortical neural cells. Therefore, shape and orientation of the stimulation coil play a critical role in determining the distribution and focus of the induced electric field in the brain. Circular coils produce a wide area of stimulation with less precise focusing, making them suitable for peripheral stimulation or treatments targeting larger cortical regions (11). Figure-eight coils provide better focusing with a smaller stimulation area, allowing precise targeting of cortical functional areas. H-shaped coils penetrate deeper into brain tissue but provide less precise focusing, making them ideal for stimulating deeper brain nuclei (12). Double-cone coils provide high-intensity stimulation to deeper regions but are prone to extensive electric field interference in shallow areas (**Figure 1C**). They are often used to stimulate motor cortex regions that control lower limb muscles. Coil orientation is also critical because it determines the direction of the induced current, which preferentially activates neurons with axons aligned along the electric field. This orientation directly influences cortical excitability. For example, in motor cortex stimulation, MEP latency is longer when TMS is applied in the anterior-posterior direction compared to the posterior-anterior direction (13). By carefully selecting and positioning the coil, clinicians and researchers can optimize TMS for specific therapeutic and diagnostic applications.

### Stimulus frequency and intensity

The frequency of TMS pulses plays a key role in modulating cortical excitability. Low frequency stimulation (below 1 Hz) reduces cortical excitability through long-term inhibitory effects, whereas high frequency stimulation (above 5 Hz) increases excitability through long-term excitatory effects (**Figure 1D**). The selection of an appropriate frequency is crucial for the treatment of brain disorders with different symptom profiles, as it directly influences clinical outcomes (14). Stimulus intensity is typically measured as a percentage of resting motor threshold (rMT), which is the percentage of maximum stimulator output required to evoke a MEP. A higher MT indicates a greater energy requirement for activation and is thought of as a basic indicator of readiness of the motor cortex to depolarize. In healthy children, rMT is the highest in infancy and declines through childhood, reaching adult levels at approximately age 12 years (15). In healthy adults, there is a significant asymmetry in their rMT, with lower excitability in the right hemisphere compared to the left. Patients with MDD also show significant asymmetry in rMT, but unlike healthy volunteers, their left hemisphere excitability is lower than the right; this asymmetry is no longer present in recovered patients (16).

### Pulse waveform and duration

The waveform and duration of TMS pulses significantly influence their neural effects and clinical applications. Monophasic pulses are characterized by a single excitatory or inhibitory effect, making them more selective in neural regulation. However, they require more energy than biphasic pulses and are generally limited to low-frequency stimulation (17). In contrast, biphasic pulses, which result from the combined excitatory and inhibitory effects, are widely used in TMS due to their higher amplitude and longer duration of action. Multiphasic pulses, with multiple cycles within a single stimulation, show increased efficacy in activating corticospinal neurons as the number of pulse cycles increases within a certain range (18). In addition, different pulse waveforms can target different groups of neurons, resulting in specific changes in neurophysiological indicators (19, 20). In spTMS, increasing pulse duration decreases both rMT and active motor threshold (aMT) without altering the regulation of motor cortex excitability. In rTMS, increasing pulse duration increases corticospinal excitability and modulates the activation of specific inter-neuronal circuits, thereby influencing therapeutic outcomes.

## TMS in brain function assessment

### Measurement of MEP

**Figure 2:**
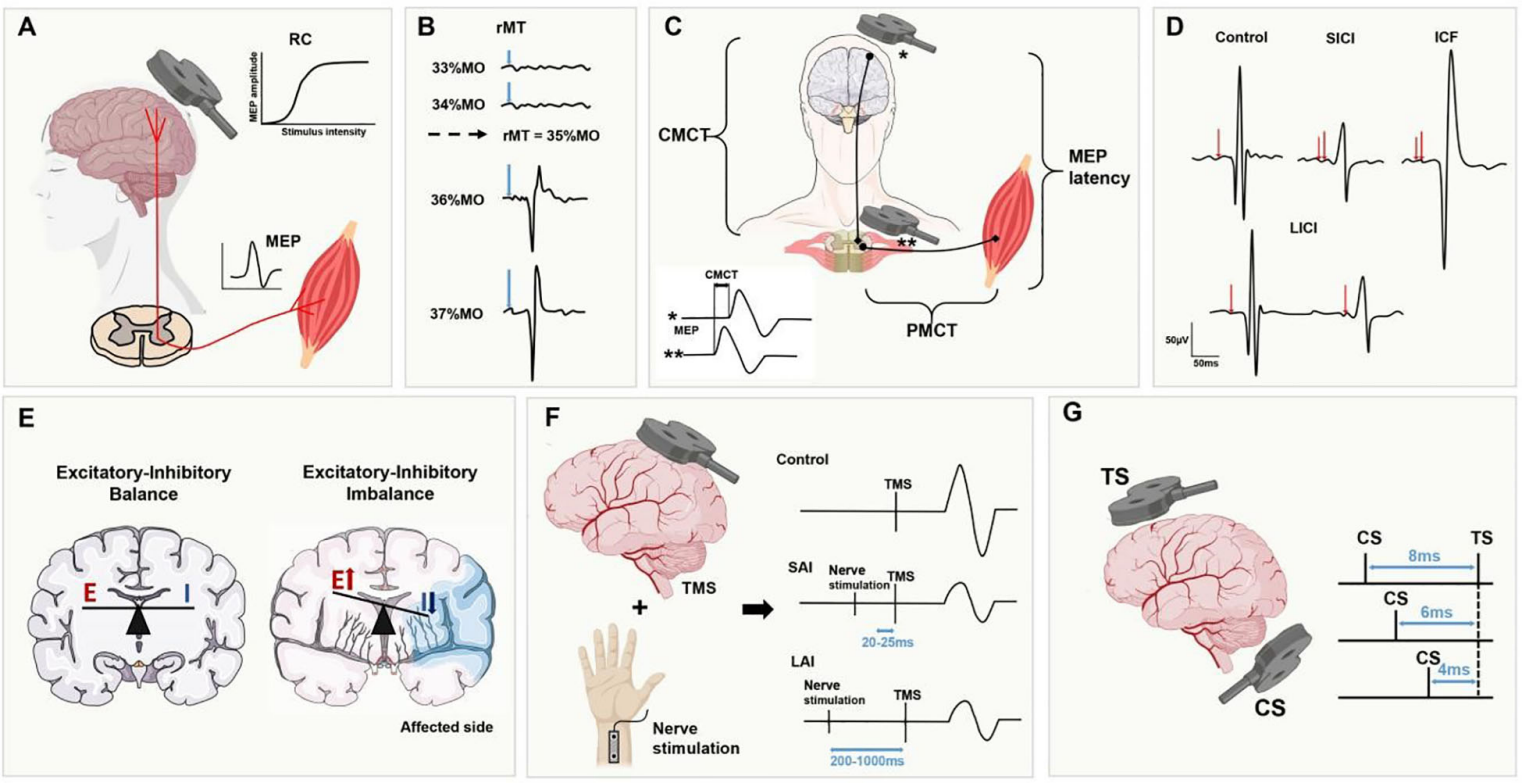
The TMS-based neurophysiological measurements. **(A)** Schematic diagrams of MEP and RC. **(B)** Schematic diagrams of rMT. **(C)** Schematic diagrams of computed CMCT. **(D)** Schematic diagrams of SICI, ICF, and LICI. **(E)** Schematic diagram of the inter-hemispheric equilibrium inhibition model. **(F)** Schematic diagrams of SAI and LAI. **(G)** Schematic diagrams of CBI. CMCT, central motor conduction time; CS, conditioning stimulus; ICF, intracortical facilitation; LAI, long-latency afferent inhibition; LICI, long-interval intracortical inhibition; MEP, motor evoked potential; RC, recruitment curve; rMT, resting motor threshold; SAI, short-Latency afferent inhibition; SICI, short-Interval intracortical inhibition; TS, test stimulus.

The MEP is the composite muscle action potential recorded from the corresponding muscles following activation of brain neurons in the motor cortex by weak currents induced by alternating magnetic fields from a TMS device at an appropriate intensity, which reflects the integrity and synchrony of the corticospinal tract (**Figure 2A**). Usually, targeted muscles are those that are relatively independent and easy to be detected by means of electromyography (EMG), such as the first dorsal interosseous muscles. The location in the motor cortex where a stable maximum MEP wave amplitude can be achieved and sustained with a minimal threshold stimulation is defined as the so-called “motor hotspot” and used to determine the target of TMS therapy (21). It should be noted that MEP is significantly influenced by functional state of the brain (22).

When TMS stimulates the cortex, the time for the excitation to pass from the cortex to the target muscle is called the latency of MEP. The amplitude of MEP waves generated after this latency period reflects the corticospinal neurons activation level, which is regulated by a complex network of dopaminergic, glutamatergic, and other neurotransmitter systems (23). Parameters such as MEP latency and wave amplitude of muscles can be used to create detailed functional maps of the motor cortex region, such as that of the pharyngeal constrictor muscle (24). Of note, factors such as age, spinal cord lesions, medications, and TMS stimulation patterns can significantly influence MEP wave amplitude. The corticomotor plasticity can be assessed as the post-TMS percent change (%Δ) in MEP amplitude elicited by spTMS. A meta-analysis claimed that iTBS on the motor cortex increases MEP but has no effect on short interval cortical inhibition (SICI) or intracortical facilitation (ICF) (25). In a recent study, the 10 Hz MEP change predicted a greater improvement on the Beck Depression Inventory II (BDI-II) and Hamilton Depression Rating Scale (HAM-D) in patients with MDD (26).

### Measurement of MT

The concept of MT is defined as the minimum stimulation intensity that reliably induces target muscle responses under specific conditions. This threshold serves as an indicator of corticospinal excitability, influenced by various factors including pulse waveforms and excitatory/inhibitory transmission at corticospinal synapses (27). The rMT is defined as the minimum stimulation intensity that elicits at least 5 MEP wave amplitudes of ≥ 50 µV in 10 consecutive stimuli while the target muscle is at rest, reflecting the excitability of the cell membrane of motor neurons (**Figure 2B**). In contrast, the aMT refers to the minimum stimulation intensity required to elicit at least 5 MEP wave amplitudes of ≥ 200 µV in 10 consecutive stimuli while the target muscle is lightly contracted. TMS may modulate cortical excitability through effects on neural synaptic plasticity and intrinsic excitability. And the effect of TMS in psychiatric conditions is mediated by altering cortical excitability in nodes of functional neural networks regulating mood and cognition (28).

### Measurement of central motor conduction time

The central motor conduction time (CMCT) is the nerve conduction time from the cerebral cortex to the alpha motor neurons in the anterior horn of the spinal cord, reflecting the conduction velocity of the motor center (**Figure 2C**). CMCT can be applied for the diagnosis of spinal cord diseases, including the localization of lesions and the determination of the degree of spinal cord compression. Prolonged CMCT indicates slowed conduction of cortico-spinal fiber bundles, commonly seen in diseases such as multiple sclerosis (MS) and amyotrophic lateral sclerosis (ALS) (29, 30). It is worth noting that CMCT is influenced by a subject’s muscle movement status, age, height, and limb length.

### Measurement of cortical silence period and ipsilateral silence period

The cortical silence period (cSP) is a period of electrical silence following a MEP in the electromyographic signal recorded from a muscle. The early components of cSP reflect spinal cord inhibition, while the late components are indicative of cortical inhibition (31). The duration of cSP exhibits an S-shaped relationship with the intensity of stimulation. An increase in MEP amplitude has been observed to extend cSP, and the ratio of cSP/MEP amplitude is used to exclude the interference of cortical excitability (32). Although cSP inhibition is thought to be mediated by Gamma-aminobutyric acid (GABA), particularly by GABA_B receptors within the primary motor cortex (M1) (33), it may also be influenced by dopaminergic transmission as evidenced by the observation that clozapine prolonged cSP in patients with schizophrenia (34).

The ipsilateral silence period (iSP) is a well-known neurophysiological TMS measure, reflecting the interruption of an ongoing ipsilateral muscle activity by stimulating M1 with a single TMS pulse. It is thought to be mediated by transcallosal inhibitory neurons from the stimulated (active) to the non-stimulated M1 and is a marker of the functional integrity of callosal motor fibers (35). The quantitative assessment of iSP involves the measurement of its duration, depth, or area to evaluate the degree of inter-hemispheric inhibition. Reduction of transcallosal inhibition has been reported in patients with ALS but the electrophysiological findings seem not to be associated with a decrease of fractional anisotropy (FA) in the motor region of the corpus callosum (CC) (36).

### Assessment of short- and long-interval cortical inhibitions and facilitations

Short interval cortical inhibition and facilitation (SICI and SICF), long interval cortical inhibition and facilitation (LICI and LICF) can be measured by sequentially applying conditioned and test stimuli (CS and TS) to the ipsilateral cerebral hemisphere using TMS (**Figure 2D**). These indicators are commonly used to study the interaction between hemispheres and evaluate the excitability of cortical circuits. SICI is achieved by applying a sub-threshold CS followed by a supra-threshold TS at an interval of 1–6 ms (37). SICI is positively correlated with working memory performance. SICF is achieved with a supra-threshold CS followed by a supra-threshold TS at intervals of 1–6 ms. SICF is more sensitive to the direction of C while SICI is more affected by CS intensity (38). It is worth noting that SICF may interfere with SICI measurements (39). A majority of studies show reduced SICI in children with ADHD, reporting stronger associations with reduced cortical inhibition among the more hyperactive/impulsive children (40, 41).

LICI is achieved by applying a supra-threshold CS, followed by a supra-threshold TS after a 50–200 ms interval (42). Different intervals can induce varying inhibitory effects, possibly involving distinct neuronal mechanisms. In a recent study, schizophrenia patients showed more LICI as compared to healthy and depressed subjects (43). This differs from the results from a large group comparison that observed less LICI in patients with schizophrenia (44). One possible explanation for different results may be different study populations. All patients with schizophrenia in the study by (43) were on antipsychotic medication, and the dose correlated with LICI, suggesting that unmedicated schizophrenia entails less LICI while antipsychotic medication increases LICI. On the other hand, higher antipsychotics dose could reflect more pronounced symptomatology, which may in itself affect cortical excitability. In addition, ICF is achieved by a sub-threshold CS followed by a supra-threshold TS at an interval of 8–30 ms. ICF is quantified as the ratio of MEP amplitudes evoked by ppTMS to those by spTMS, the larger the ratio, the stronger the excitatory interneuron network function within the cortex.

### Assessment of interhemispheric inhibition

Interhemispheric inhibition (IHI) is a neurophysiological parameter reflecting the inhibitory signals transmitted via CC between hemispheres (**Figure 2E**). It can be assessed by analyzing the effect of a CS to one M1 on the MEP amplitude evoked by a TS to the opposite M1 at intervals between 6–50 ms (45). IHI is classified into short-latency IHI and long-latency IHI, which involve distinct neural circuits and exhibit additive effects. The intensity of IHI is typically quantified as the ratio of the mean MEP amplitude evoked by ppTMS to that evoked by spTMS. Of note, IHI is influenced by factors such as facial emotional expressions, age, and motor imagery, highlighting its sensitivity to individual and contextual variations. In a recent study, bifocal transcranial alternating current stimulation (tACS) over the primary sensorimotor cortices increased IHI and improved bimanual dexterity in healthy participants (aged 18 to 37 years) (46).

### Assessment of short- and long-latency afferent inhibitions, and cerebellar brain inhibition

Short-latency afferent inhibition (SAI) is characterized by suppression of MEP amplitude when peripheral nerve stimulation precedes TMS by 20–25 ms (47). SAI is markedly reduced in central cholinergic degenerative diseases, making it a valuable early biomarker for diseases such as Alzheimer’s disease (AD) and Parkinson’s disease (PD) (48, 49). In research with schizophrenia patients, N100 on TMS-evoked potentials (TEPs) was significantly attenuated with dorsolateral prefrontal cortex (dlPFC) -SAI, whereas P180 TEP was significantly increased with M1-SAI. Furthermore, modulation of N100 TEP by dlPFC-SAI significantly correlated with executive function of the patients (50). Long-latency afferent inhibition (LAI) refers to the suppression of MEP by a peripheral nerve stimulation for 200–1000 ms prior to TMS (**Figure 2F**) (51). The intensity of SAI and LAI increases with increasing stimulus intensity, although their associations with age, gender, sensory processing, and motor tasks remain controversial.

Cerebellar-brain inhibition (CBI) is the suppression of MEP amplitude by a conditioning TMS pulse applied to the cerebellum at 4–8 ms prior to a test pulse to the M1 (**Figure 2G**) (52). It is achieved via cerebellar-thalamic-cortical pathways and used to evaluate cerebellar connectivity with the motor cortex. Factors such as aging and fatigue may reduce CBI (53, 54). For reliable and tolerable CBI measurement, a double cone coil at 60% maximum stimulus power is recommended (55). Importantly, CBI demonstrates high specificity by inhibiting only the target muscles without affecting surrounding muscles, making it a useful tool in the treatment of hand stiffness (56). CBI has potential applications in the diagnosis and assessment of conditions such as essential tremor and PD.

### Neuroimaging-based assessment

The combined application of TMS and neuroimaging-based assessment can improve the efficiency of neurophysiological detection and help to realize the accurate positioning of TMS targets and promote individualized treatment. For example, fMRI was used to detect blood oxygen level-dependent (BOLD) signal changes while spTMS was employed to stimulate the cortical projection area corresponding to the inferior anterior cingulate cortex (sgACC) or basolateral amygdala (BLA) of participants in a recent study. Thereby, the authors generated a reliable functional connectivity map between cortical and subcortical brain regions, thus realizing the causal inference of TMS effect (57). Of note, the time resolution of fMRI is not high enough to capture immediate blood oxygenation (oxy-Hb) effects resulted from spTMS in this kind of cases. Differently, functional near-infrared spectroscopy (fNIRS) is highly suitable for simultaneous application during rTMS treatment, despite its limited spatial resolution. For example, Struckmann et al. demonstrated the modulation of dlPFC functional connectivity after intermittent theta-burst stimulation in depression by combining findings from fNIRS and fMRI (58). In another study, TMS and fluorodeoxiglucose [^18^F]FDG-PET were combined to evaluate the ability of TMS-MEPs in delineating the neurophysiological upper motor neuron damage, and to determine the relationship between TMS-MEPs and [^18^F]FDG-PET measures of neural dysfunction (59). In summary, the combined application of TMS and multiple neuroimaging techniques can achieve multimodal verification of the causal effects of neural circuits from different spatial-temporal scales and biological dimensions, thereby promoting individualized treatment.

## The therapeutic effects of TMS on major mental disorders

In November 2014, *Clinical Neurophysiology* published a consensus paper on the therapeutic use of rTMS in neuropsychiatric diseases (60). During the following six years, the same group updated their recommendations for the applications of rTMS in clinical practice (61). According to the updated version of consensus paper, rTMS can produce significant clinical improvement in various neurological and psychiatric disorders.

### The therapeutic effects on patients with depression

The efficacy and safety of TMS in the treatment of depression have been explored in numerous studies. In clinical practice, the exact therapeutic outcomes may vary depending on factors such as the specific stimulation site, heterogeneity of patients’ pathogenesis and their symptom profile. Low-frequency stimulation of the right dlPFC or high-frequency stimulation of the left dlPFC are common approaches for TMS therapy of depression. Among these, the left dlPFC is widely considered the most effective site for treating MDD (62). This brain region shows negative functional connectivity with the sgACC. To locate dlPFC, the “5 cm rule” and Beam F3 method are widely used. Specifically, the “5 cm rule” is a simple landmark-based method. It first accurately identifies the motor hotspot over the M1, and then the target stimulation site is determined by moving 5 cm anteriorly along the scalp from this hotspot. Although this method is easy to implement, it suffers from high variability due to individual differences in head circumference and underlying neuroanatomy. In contrast, the “Beam F3” method performs standardized heuristic localization based on the F3, Fz, and Cz scalp coordinates derived from the 10–20 electroencephalography (EEG) system, achieving higher targeting accuracy. Recently, researchers have developed personalized targeting approaches for TMS based on the interaction between the dlPFC- subgenual cingulate cortex (SGC)-vagal nerve (VN) utilizing a neuro-cardiac-guided method to achieve individualized localization. In a more recent study by Croarkin et al., stimulation of the right dlPFC (120% rMT, 1 Hz, 360 pulses) followed by stimulation of the left dlPFC (120% rMT, 20 Hz, 1200 pulses) once daily for five days significantly alleviated depression and anxiety symptoms in adolescents with treatment-resistant depression (TRD) (63). In elderly patients with depression, stimulation of the left dlPFC at 110% of MT was found to significantly improve response and remission rates compared to 80% MT stimulation (64).

A meta-analysis demonstrated the efficacy and safety of bilateral rTMS in patients with TRD, and the results suggest that bilateral TBS may be the most effective neuromodulation strategy for treating TRD (65). However, a more recent randomized controlled trial found no statistically significant difference in the clinical efficacy between bilateral TBS and bilateral rTMS treatment, both of which maintained their effects for up to 26 weeks (66). Of note, Stanford Accelerated Intelligent Neuromodulation Therapy (SAINT), using 90% rMT (adjusted according to cortical depth), 1,800 pulses per session, with a 50-minute interval, administered 10 sessions daily for 5 consecutive days, has shown significant symptom alleviation in TRD patients, although the durability of its effects warrants further investigation (66, 67). This therapy has not been associated with significant cognitive side effects, even benefit cognitive improvement in elderly patients with depression (68). Interestingly, different symptom clusters of depression responded differently to stimulation at various TMS sites. For instance, the peak target for anti-anxiety effects has been identified at the left dlPFC (MNI coordinates [−37, 22, 54]) and the dorsomedial prefrontal cortex (dmPFC) (MNI coordinates [18, 37, 55]), while the peak target for alleviating irritability is located at MNI coordinates [−32, 44, 34] (69). Moreover, individualized functional connectivity (FC)-guided rTMS was demonstrated to be an effective anti-depressant treatment. For example, 52.5% of TRD patients showed symptom remission following treatment with individualized rTMS guided by the peak FC in the dlPFC seeded from the sgACC, which is considered an effective region for rTMS treatment of depression (70, 71).

### The therapeutic effects on patients with BD

Patients with BD exhibit complex clinical presentations, characterized by alternating episodes of depression, (hypo)mania, and mixed states. The higher heterogeneity and greater volatility of the disorder in these patients are related to the more diverse therapeutic effects of TMS in treating BD compared to patients with depression. Therefore, TMS is predominantly used as an adjunctive therapy alongside pharmacological treatment in BD. In a double-blind, randomized, parallel group, sham-controlled clinical trial, 20 sessions of TMS with an H1 coil at 18 Hz, 2 seconds, and 120% MT significantly alleviated depressive symptoms in patients with treatment-resistant bipolar depression (TRBD) who had received adequate pharmacotherapy (72). Of note, treatment protocol used in TMS influences symptom improvement and patient prognosis. For instance, in acute BD, iTBS targeting the left dlPFC did not demonstrate antidepressant effects, and some patients even experienced a switch to hypomania (73), whereas a meta-analysis of rTMS treatment for acute BD concluded that low-frequency stimulation of the right dlPFC may lead to more significant clinical outcomes (74). Kazemi et al. (75) proposed that bilateral stimulation may be more effective than unilateral stimulation. However, a randomized controlled trial with a parallel design using bilateral TMS (1 Hz low-frequency stimulation of the right dlPFC and 10 Hz high-frequency stimulation of the left dlPFC) in BD patients did not show significant clinical improvements on depression rating scale scores or remission rates (76). Thus, the optimal protocol and efficacy of bilateral TMS in BD require further investigation and validation. There is limited clinical data on the use of TMS in the treatment of bipolar mania and patients in the maintenance phase, and no definitive conclusions can be drawn (77). As for mixed-state BD, low-frequency (1 Hz) stimulation of the right dlPFC in 40 patients resulted in statistically significant improvements on both depressive and manic symptoms (78). However, in another double-blind, randomized controlled trial, bilateral TBS targeting the dlPFC did not achieve clinically meaningful effects in mixed-state BD patients (79).

### The therapeutic effects of TMS on patients with schizophrenia

Schizophrenia is characterized by three major symptom clusters: positive symptoms (hallucinations, delusions, and disorganized speech and behavior), negative symptoms, and cognitive impairments. While pharmacological treatments for positive symptoms are effective, there is a paucity of effective drug therapies for negative symptoms and cognitive dysfunction. In recent years, the application of TMS has been progressively explored as a treatment for schizophrenia, with TBS showing significant potential in improving negative symptoms and cognitive function associated with the disorder (80). For example, iTBS targeting the vermis of the cerebellum in schizophrenia patients, with two daily sessions over a five-day period, improved connectivity between the right dlPFC and cerebellar networks, leading to significant improvements in negative symptoms of schizophrenia (81). Moreover, TMS targeting the frontal and temporal lobes of schizophrenia patients, improved specific cognitive functions, including working memory and attention (82). However, due to the complexity and interrelated nature of schizophrenia symptoms, the efficacy of TMS in symptom management has yielded inconsistent research findings and still requires further evidence-based validation. A meta-analysis of randomized controlled trials showed that high-frequency stimulation (20–50 Hz) at 110% MT targeting the left prefrontal cortex (PFC) for more than three weeks can improve negative symptoms, but it may also exacerbate positive symptoms (83). Some studies have suggested that TMS targeting the temporoparietal cortex may improve refractory hallucinations and positive symptoms of schizophrenia, although the results are not sufficiently reliable (84, 85). A recent meta-analysis also denied the efficacy of TMS in improving cognitive function in schizophrenia (86).

### The therapeutic effects of TMS on patients with ADHD

As early as 2003, Acosta and Leon-Sarmiento (87) explored the available evidence that makes rTMS a rational therapeutic possibility for patients with ADHD, and the authors advocated clinical trials to investigate efficacy, safety, and clinical utility of rTMS for ADHD patients. Since then, rTMS, as a non-invasive neuromodulatory intervention, has been applied to the treatment of ADHD in both adult and child/adolescent populations (88, 89). Across clinical studies in ADHD, TMS protocols have varied widely in stimulation frequency, intensity, coil type, and treatment duration, contributing substantially to variability in reported outcomes. Most studies have applied high-frequency rTMS (10–20 Hz) over prefrontal regions at intensities of 100–120% of rMT, delivered across 10–20 sessions over 2–4 weeks. Both focal figure-8 coils and deep TMS coils have been used, with the latter typically targeting bilateral prefrontal regions (89, 90).

In 2010, positive effects of rTMS on attention in ADHD subjects were reported in a randomized controlled pilot study (90), while Weaver et al. (91) tested the safety and efficacy of TMS in young persons with a diagnosis of ADHD. TMS was found to be safe, with no serious adverse events. Following the above studies, Paz et al. (92) reported no differences in clinical outcomes between the actual dTMS and sham groups of adult ADHD patients, while another study reported that three weeks of daily high-frequency (18 Hz) stimulation was safe and resulted in significant improvement of symptoms in adult ADHD patients (93). Similarly, a double blind, randomized clinical trial reported significant improvements on the CAARS (the Conners Adult ADHD Rating Scale, self-report) inattention/memory sub-scale, as well as increased activations in the right dlPFC, although the study did not show improvement in the primary endpoints (94). In a recent study, low-intensity magnetic stimulation of the dlPFC improved response inhibition (reduce errors) even in a single-session intervention of ADHD individuals (95). A recent meta-analysis encompassing 8 samples from 7 studies demonstrated that rTMS significantly improved ADHD symptoms compared to control conditions (96). In another meta-analysis, Fu et al. (89) concluded that TMS significantly improved the inattention, hyperactivity/impulsivity, and total symptom scores in ADHD patients with minor adverse events.

### The therapeutic effects of TMS on patients with ASD

ASD is a group of neurodevelopmental disorders, which mainly occur in childhood and affect their whole life with social communication impairment and restricted interests/repetitive behaviors. According to a recent systematic analysis of the global status of mental disorders, the global age-standardized prevalence of ASD was 369.4 cases per 100,000 people in 2019, with a significant male predominance (97). Although early psychological/behavioral intervention, complementary therapy, or drug treatment can improve some symptoms of ASD, these treatments cannot completely correct the core symptoms of ASD (social communication impairment). In the past 20 years, due to the development of medical physics and medical physiology, neuromodulation techniques have come to the fore and been widely used in preclinical and clinical research of treatment for ASD (98).

In an early sham-controlled study involving 28 adults with Asperger’s disorder (15 real, 13 sham), 10 daily sessions of deep 5 Hz-rTMS (1500 pulses/session) significantly reduced social relating symptoms (especially self-oriented anxiety during difficult and emotional social situations) in the rTMS group, but not in the sham group (99). Another study compared the effect of 1 Hz-rTMS of the left dlPFC (F3 site, 20 daily sessions, 1500 pulses/session, 90% of rMT) to that of anodal transcranial direct-current stimulation (tDCS) over the same target in 24 children with ASD. The severity scores of autism related symptoms in both groups of patients showed significant improvement, while there was no significant difference in the improvement effect caused by rTMS and tDCS (100). In a recent pilot study with 20 ASD children (ages 2–13), a 15-week protocol involving extremely low-frequency electromagnetic field (ELF-EMF) significantly improved externalizing, with particular improvement in attention and behavioral problems of the patients (101). In another study, LF-TMS targeting the dlPFC in children with ASD effectively improved their sleep status, and significant improvement was achieved after 6 weeks (30 sessions) of treatment (102). Similarly, a randomized controlled trial (RCT) study presented preliminary evidence on the effect and safety of alpha rhythm-guided rTMS in improving subjective sleep difficulties in children with ASD, with effects lasting up to four months post-intervention (103). Moreover, an intervention combining low-intensity TMS with conventional therapies significantly improved the personal, social, motor, cognitive, and communication abilities of all 35 participants with ASD (6 females and 29 males), without any adverse effects (104). In a latest systematic review and meta-analysis of randomized controlled trials published up to April 25, 2025, rTMS improved social communication, repetitive and abnormal behaviors in children and young adults with intellectually capable ASD (105).

## The molecular mechanisms underlying the therapeutic effects of TMS on mental disorders

### TMS regulates the expression of neurotransmitters in the brain

#### Dopamine

Dopaminergic signaling mediates several neuropsychological processes, including reward, cognition, and memory. High-frequency rTMS stimulation of frontal brain regions has been shown to enhance DA release in the dorsal hippocampus, nucleus accumbens (NAc), and dorsal striatum, potentially aiding in the treatment of emotional disorders and PD (106, 107). Evidence suggests that rTMS at a maximum power of 60% produces the highest DA release, indicating a possible inverted U-shaped relationship between TMS intensity and striatal DA release (108). Of note, high-frequency stimulation appears to be more effective than low-frequency stimulation in increasing DA levels (109). When applied to the motor cortex and PFC, TMS reduces DA receptor availability in the ipsilateral putamen and caudate nucleus, reflecting increased DA release (110, 111).

#### Serotonin

In the central nervous system (CNS), 5-HT modulates a broad spectrum of functions, including mood, cognition, anxiety, learning, memory, reward processing, and sleep. Deficits in the serotonergic system involve in various mental disorders, such as depression, schizophrenia, and autism. rTMS has been shown to affect mood in health and disease as reviewed above. In a previous PET study, acute prefrontal cortex TMS in healthy volunteers increased 5-HT release in the right posterior cingulate cortex, but not cortical or sub-cortical regions (112). In another PET study on patients with depression, high-frequency rTMS (HF-rTMS) showed significant antidepressant effects, with changes in symptom severity positively correlated with change in 5-HT2A receptor binding in bilateral dlPFC but negatively correlated with right hippocampus receptor binding (113). In patients with generalized anxiety disorder (GAD), bilateral low-frequency rTMS markedly decreased Hamilton anxiety rating scale (HARS) while increased the levels of serum brain derived neurotrophic factor (BDNF) and 5-HT (114). Consistent with the above human studies, rTMS down regulated 5-HT2 receptors in the frontal cortex but had no effect on the other brain areas of rats (115). Related to this, electrical stimulation of the medial PFC produced current-dependent increases in limbic 5-HT output in both urethane-anesthetized and behaving rats. Of note, no effects on 5-HT levels were seen following comparable stimulation of either the lateral parts of the PFC, the medial precentral area, the M1 or the parietal cortex. This regional specificity of the effect of medial PFC stimulation on limbic 5-HT output suggests that activation of this particular area might play a crucial role in such antidepressant treatments as electroconvulsive therapy (ECT) and TMS (116). Furthermore, rTMS (5/10 Hz, 0.84/1.26 T) ameliorated depressive-like behaviors, while increased 5-HT, DA and NE levels in the PFC of chronic unpredictable stress-treated rats (117).

#### Glutamate

Glutamate is an excitatory neurotransmitter acting on N-methyl-D-aspartate (NMDA) and α-amino-3-hydroxy-5-methyl-4-isoxazolepropionic acid (AMPA) receptors, and able to mediate the induction and maintenance of short- and long-term synaptic plasticity through vesicle trafficking or lateral diffusion across the postsynaptic membrane. Related to this, NMDA receptor agonists have been shown to potentiate TMS-induced changes in MEP, suggesting a role for NMDA receptors in TMS-induced synaptic plasticity (118). In two animal studies, iTBS was found to alter NMDA receptor subunit composition in the striatum, with increased expression of GluN2A and GluN2B subunits (119, 120). Moreover, high-frequency (10 Hz) stimulation of mature hippocampal CA1 pyramidal neurons induced long-term increases in glutamatergic synaptic strength, activity-dependent up-regulation of GluA1-containing AMPA receptors, and structural remodeling of dendritic spines in mouse organotypic hippocampal slice cultures (121).

#### Gamma-aminobutyric acid

In the brain, there is a dynamic balance between excitation and inhibition, with glutamate serving as the primary excitatory neurotransmitter and GABA as the primary inhibitory neurotransmitter. Abnormalities in GABA levels have been associated with several mental disorders (122, 123). Using proton magnetic resonance spectroscopy [(1)H-MRS], one study found that TMS treatment increased GABA levels in the medial prefrontal cortex (mPFC) of patients with major depression by 13.8% (124). Similarly, 1 Hz rTMS increased GABA levels in the dlPFC in patients with chronic insomnia (125).

### TMS suppresses neuroinflammation in the brain

Inflammation is a risk factor for depression given that patients with autoimmune and infectious diseases are more likely to develop depression. Interestingly, rTMS was shown to decrease interleukin-1(IL-1) and tumor necrosis factor-alpha (TNF-α) but increase serum BDNF in elderly patients with TRD (126). Moreover, rTMS enhanced antidepressant- and anxiolytic-like effect via nuclear factor-E2-related factor 2-mediated anti-inflammation mechanism in rats (127). In support of this anti-inflammation mechanism, low-intensity rTMS treatment of astrocytes alters gene expression and protein levels related to calcium signal transduction, inflammatory molecules, and neuroplasticity (128). Moreover, 10 Hz rTMS decreased TNF-α but increased the anti-inflammatory mediator IL-10, as well as reduced neuronal apoptosis in rats with cerebral ischemia reperfusion injury (CIRI) (129). It was also reported that high frequency rTMS exerts anti-inflammatory and neuroprotective effects by regulating the endogenous cannabinoid system and inhibiting astrocyte proliferation (130). Related to this, iTBS was shown to significantly ameliorate levels of IL-1β, IL-17A, TNF-α, and IFN-γ in a mouse model of CIRI by inhibiting pro-inflammatory M1 activation, enhancing anti-inflammatory M2 activation, and restoring the balance of M1/M2 microglial phenotypes (131).

### TMS promotes the production of BDNF

BDNF is a vital protein with functions of promoting the survival, growth, and maintenance of neurons in CNS. It has been linked to brain disease and thought to play fundamental role in synaptic plasticity. Specifically, BDNF has emerged as an important player in the pathogenesis of MDD and a potential diagnostic biomarker for this brain disease (132).

In a randomized case–control study, the serum levels of BDNF and glial cell line-derived neurotrophic factor (GDNF) were initially lower in MDD patients compared to healthy subjects. Following a rTMS protocol (10 Hz, a total of 37.5 min, 4 s of stimulation time, and 26 s of latent time, 20 sessions every weekday for a month), the levels of the two neurotrophic factors were significantly increased alongside with decreased Montgomery–Asberg Depression Rating Scale (MADRS) (133). Similarly, when rTMS was applied simultaneously with agomelatine (daily dose ranging from 25 to 75 mg based on clinical response and side effects for 8 weeks), a reduction in the Hamilton Depression Rating Scale (HDRS) score was observed. And the clinical outcome was accompanied by increased serum levels of BDNF and norepinephrine (134). In another clinical study focusing on middle-aged and elderly patients with MDD, rTMS in combination with escitalopram (5–20 mg/d) showed a more effective antidepressant effect compared to oral escitalopram alone. Additionally, this combined protocol resulted in increased serum BDNF levels (135). In TRD patients, rTMS (20 Hz,2-s duration, administered 20 times at 30-s intervals, at 100% of the motor threshold, five days a week for four weeks) produced an antidepressant effect, which was associated with the normalization of serum BDNF and GDNF levels (136). Moreover, researchers applied the rhythmic low-field magnetic stimulation (LFMS, 20 min per session,5 sessions per week, for 6 weeks) in the form of rhythmic alpha stimulation (RAS) or rhythmic delta stimulation (RDS) for patients with MDD. Interestingly, the clinical evaluation of the patients showed a significantly higher effect size in the RDS group, whereas the increase in serum BDNF levels was more pronounced in the RAS group. The authors proposed that a low baseline serum BDNF level may be a predictive biomarker for the efficacy of rhythmic magnetic stimulation (137).

### TMS exhibits anti-oxidant effects

There is increasing evidence for the anti-oxidative stress action of TMS as shown in preclinical and clinical studies. In patients with multiple sclerosis (MS), treatment with 1 Hz rTMS decreased plasma oxidative stress levels and improved neuropsychological function of the patients (138). In 3xTg-AD mice, 25 Hz rTMS reduced hippocampal Aβ1–42 levels, ameliorated oxidative stress while improved cognitive function of the animals (139). Moreover, high frequency (60 Hz) TMS significantly reduced oxidative stress levels in cortical synapses and improved cognitive function in Wistar rats subjected to 3-nitropropionic acid-induced neurotoxicity (140, 141). In 6-hydroxydopamine-induced mouse model of PD, 21 days of iTBS treatment increased antioxidant parameters in the caudate nucleus and substantia nigra pars compacta, decreased serum oxidative markers, and increased antioxidant levels, highlighting the potential neuroprotective effects of iTBS (142). In a recent randomized case-control study, rTMS treatment reduced oxidative stress and restores thiol-disulfide balance in MDD patients (133).

### TMS regulates the expression of genes associated with brain plasticity and apoptosis

Previous studies have demonstrated that rTMS suppresses the apoptosis and apoptotic pathways, thereby exerting neuroprotective effects on neurons in an affected brain (143). Furthermore, research has shown that rTMS therapy can specifically up-regulate the expression of Bcl-2 family genes and down-regulate the expression of Bax family genes, promoting the balance of the Bax/Bcl-2 ratio, thereby exerting neuroprotective effects in preclinical and clinical studies (144, 145). In a middle cerebral artery occlusion rat model, 10 Hz rTMS treatment markedly upregulated Bcl-2 expression and decreased the levels of Bax and TUNEL-positive cells in the ischemic hippocampus (146). In an animal model of focal cerebral ischemia, rTMS rescued DNA damage and reduced the expression of genes related to inflammation and axonal development (147). In an AD mouse model, rTMS (1 and 10 Hz) treatment decreased apoptosis, as reflected by enhanced Bcl−2 expression and decreased levels of Bax and cleaved caspase−3 (148).

**Figure 3:**
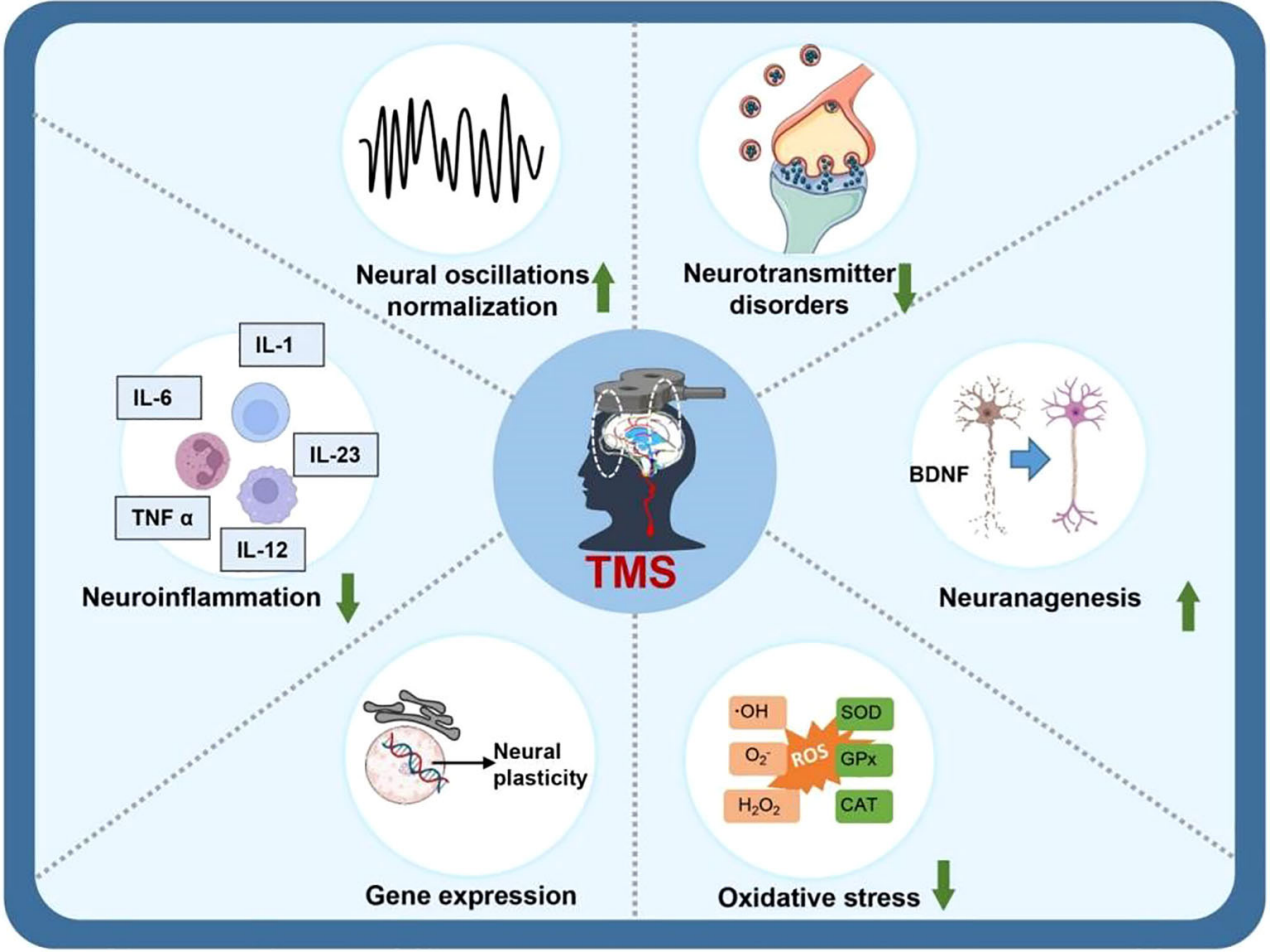
Molecular mechanism of TMS. BDNF, brain-derived neurotrophic factor; CAT, catalase; GPx, glutathione peroxidase; IL-1, interleukin 1; IL-6, interleukin 6; IL-12, interleukin 12; IL-23, interleukin 23; ROS, reactive oxygen species; SOD, superoxide dismutase; TNF-α, tumor necrosis factor α.

In summary, the molecular mechanisms underlying the therapeutic effects of TMS on neuropsychiatric disorders involve modulating neural oscillations and the release of neurotransmitters, inhibiting neuroinflammation, promoting the production of BDNF, decreasing oxidative stress, and regulating the expression of genes associated with brain plasticity and apoptosis (**Figure 3**).

To help readers more easily compare and understand the main points of this review article, we created a table to highlight the key conclusions, including major disorders, stimulation targets, clinical applications, mechanisms, and current evidence resulted from the references cited in this study (**Table 1**).

**Table 1:** The summary table with major disorders, stimulation targets, clinical applications, potential mechanisms, and current evidence.

Mental disorders	Stimulation target	Stimulus frequency	Clinical applications	Potential mechanisms	Current research evidence	References
Treatment-resistant depression	left dlPFC	10 Hz (rTMS) /50 Hz intra-burst, 5 Hz inter-burst (iTBS)	Sustained antidepressant improvements lasting up to 26 weeks.	Rebalance the reciprocal effective connectivity between the right anterior insula and left dlPFC.	A five-center, parallel, double-blind, randomized controlled trial	Morriss et al., 2024 (149)
Treatment-resistant depression	left dlPFC, right dlPFC	50 Hz intra-burst, 5 Hz inter-burst (iTBS), 50 Hz intra-burst, 5 Hz inter-burst (cTBS)	Effectively alleviate core depressive symptoms via bilateral TBS and standard bilateral rTMS. Preserve cognitive function during treatment, suitable for elderly patients.	Mimics endogenous γ–theta rhythm coupling to induce robust synaptic plasticity.	A randomized noninferiority trial	Blumberger et al., 2022 (150)
Geriatric depression	bilateral dlPFC (left dlPFC dominant)	50 Hz intra-burst, 5 Hz inter-burst (iTBS)	SANIT presents several aspects that are potentially highly beneficial for late-life depression: efficacy, rapid action, and cognition improvement.	Modulates bilateral dlPFC cortical excitability and rebalances emotional network activity.	Commentary with exploratory clinical analysis	Batail et al. (68)
Bipolar disorder	left dlPFC	18 Hz (dTMS)	Improves depressive symptoms and global functioning. Safe without inducing mood switch.	Penetrate deeper prefrontal circuits; modulate cortical excitability.	A randomized sham-controlled trial	Tavares et al. (72)
Bipolar disorder	left dlPFC	50 Hz intra-burst, 5 Hz inter-burst (iTBS)	SAINT accelerated iTBS yields significant rapid reduction in MADRS; 50% response rate and 40% immediate remission rate, up to 60% remission within 1 month; no mania switch, severe adverse events or cognitive impairment.	Modulate prefrontal-limbic circuit connectivity; normalize abnormal network anticorrelation; induce rapid synaptic plasticity.	A two-site open-label feasibility trial	Li et al. (86)
Schizophrenia	cerebellar vermis (cerebellar midline, VIIb)	50 Hz intra-burst, 5 Hz inter-burst (iTBS)	Improve medication-refractory negative symptoms in schizophrenia; target cerebellar-prefrontal circuit dysfunction.	Restore disrupted functional connectivity between right dlPFC and cerebellar midline; modulate cerebellar-prefrontal neural circuit.	A randomized sham-controlled study	Brady et al. (81)
Schizophrenia	left TPJ	specific frequency not stated(rTMS)	Adjunctive treatment for medication-refractory auditory verbal hallucinations in schizophrenia.	Modulate abnormal neural activity of left TPJ; repair disrupted auditory hallucination-related brain network.	A double-blind, randomized sham-controlled clinical trial	Hua et al., 2024 (151)
Attention deficit/hyperactivity disorder (ADHD)	bilateral prefrontal cortex	18Hz (dTMS)	Randomized sham-controlled trial showed no significant clinical differences between active dTMS and sham group on CAARS and TOVA; both groups had mild symptom improvement with prominent placebo effect.	Modulate bilateral prefrontal cortical excitability; regulate fronto-striatal circuit function; improve attention and executive control related neural activity.	A randomized double-blind sham-controlled clinical trial	Paz et al., 2017 (152)
Attention deficit/hyperactivity disorder (ADHD)	dlPFC/vlPFC/cerebellar-related cognitive network regions	>5 Hz (rTMS)/ (dTMS)	TMS significantly improves inattention and hyperactivity/impulsivity at 3–6 weeks; inattention and total symptom benefits sustain at 1-month follow-up	Modulate fronto-striatal, cingulo-frontal-parietal and fronto-cerebellar circuits; regulate neurotransmission; induce synaptic plasticity; repair prefrontal hypoactivity in ADHD.	Systematic review and meta-analysis	Fu et al. (89)
Autism spectrum disorder (ASD)	left dlPFC (BA9/BA46)	10Hz (rTMS)	Improve error monitoring/correction performance and enhance ERN amplitude in ASD patients; alleviate executive dysfunction, repetitive behaviors and social cognitive deficits.	Modulate prefrontal cortical excitability and restores excitation-inhibition (E/I) balance; induce synaptic plasticity in the cognitive control network; improve neural synchronization underlying error processing.	A randomized controlled wait-list clinical trial	Sokhadze et al., 2012 (153)
Autism spectrum disorder (ASD)	left dlPFC	1Hz (Li-TMS)	Adjunct intervention for children aged 3–7 with ASD; improve cognitive, language, motor, social and adaptive abilities; reduce neurodevelopmental delay.	Modulate cortical excitation–inhibition balance; regulate intracellular calcium signaling; promote synaptic plasticity.	A retrospective longitudinal observational study	Espinosa Mendoza et al. (104)

rTMS, repetitive transcranial magnetic stimulation; dlPFC, Dorsolateral Prefrontal Cortex; iTBS, intermittent theta burst stimulation; cTBS, continuous theta burst stimulation; dTMS, deep transcranial magnetic stimulation; SAINT, Stanford Accelerated Intelligent Neuromodulation Therapy; MADRS, Montgomery–Åsberg Depression Rating Scale; TPJ, temporo-parietal junction; vlPFC, ventrolateral prefrontal cortex; CAARS, Conners' Adult ADHD Rating Scales; TOVA, Test of Variables of Attention; Li-TMS, Low-intensity TMS.

## Concluding remarks

As a safe and effective non-invasive brain stimulation technique, TMS has immense potential in clinical research and applications. By integrating non-invasive neuroimaging techniques such as fMRI and computational modeling, researchers can now pinpoint key nodes within individual brain functional networks and tailor stimulation parameters to achieve precise neuromodulation. Parallel to this, the field is expanding its clinical applications through technological innovations, including multi-focal coils for simultaneous multi-region stimulation and novel paradigms such as burst stimulation. One particularly promising direction lies in combining TMS with cognitive behavioral therapy or pharmacotherapies during plasticity-sensitive windows, a strategy that facilitates more effective remodeling of impaired neural circuits. Moreover, the fusion of TMS and multimodal neuroimaging is expected to improve treatment targets through the “intervention observation” feedback loop, ultimately promoting sustained breakthroughs in the treatment of mental disorders.

Despite the aforementioned applications, there are some challenges in the application of TMS. First, current clinical research and applications mainly target superficial brain regions such as dlPFC. This limitation has restricted the application of TMS in the diagnosis and treatment of brain disorders involving deep brain structures. Second, the lack of disease-specific TMS treatment protocols remains a significant barrier. There are far fewer TMS protocols used to treat various brain diseases, which limits the therapeutic effectiveness of TMS. Third, there is an urgent need to develop treatment protocols that combine drug therapy and TMS to achieve synergistic efficacy in treating mental disorders while minimizing side effects. Addressing these challenges through innovative research and technological advances will further unlock the potential of TMS as a versatile diagnostic and therapeutic tool, paving the way for improved management of mental disorders.
